# Identification of Anti-TNFα VNAR Single Domain Antibodies from Whitespotted Bambooshark (*Chiloscyllium plagiosum*)

**DOI:** 10.3390/md20050307

**Published:** 2022-04-29

**Authors:** Linfei Zhao, Mingliang Chen, Xiaona Wang, Shoukai Kang, Weiwei Xue, Zengpeng Li

**Affiliations:** 1 School of Fisheries and Life, Shanghai Ocean University, Shanghai 201306, China; zhaolinfei1996@outlook.com; 2 Key Laboratory of Marine Genetic Resources, State Key Laboratory Breeding Base of Marine Genetic Resources, Fujian Key Laboratory of Marine Genetic Resources, Fujian Collaborative Innovation Centre for Exploitation and Utilization of Marine Biological Resources, Third Institute of Oceanography Ministry of Natural Resources, Xiamen 361005, China; mlchen_gg@tio.org.cn; 3 Co-Innovation Center of Jiangsu Marine Bio-Industry Technology, Jiangsu Ocean University, Lianyungang 222005, China; 4 School of Pharmaceutical Sciences, Chongqing University, Chongqing 401331, China; 202029021021@cqu.edu.cn; 5 Department of Biochemistry, Institute for Protein Design, University of Washington, Seattle, WA 98195, USA; kangsk@uw.edu

**Keywords:** whitespotted bambooshark, IgNAR, VNAR, single domain antibody, TNFα

## Abstract

Tumor necrosis factor α (TNFα), an important clinical testing factor and drug target, can trigger serious autoimmune diseases and inflammation. Thus, the TNFα antibodies have great potential application in diagnostics and therapy fields. The variable binding domain of IgNAR (VNAR), the shark single domain antibody, has some excellent advantages in terms of size, solubility, and thermal and chemical stability, making them an ideal alternative to conventional antibodies. This study aims to obtain VNARs that are specific for mouse TNF (mTNF) from whitespotted bamboosharks. After immunization of whitespotted bamboosharks, the peripheral blood leukocytes (PBLs) were isolated from the sharks, then the VNAR phage display library was constructed. Through phage display panning against mTNFα, positive clones were validated through ELISA assay. The affinity of the VNAR and mTNFα was measured using ELISA and Bio-Layer Interferometry. The binding affinity of 3B11 VNAR reached 16.7 nM. Interestingly, one new type of VNAR targeting mTNF was identified that does not belong to any known VNAR type. To understand the binding mechanism of VNARs to mTNFα, the models of VNARs-mTNFα complexes were predicted by computational modeling combining HawkDock and RosettaDock. Our results showed that four VNARs’ epitopes overlapped in part with that of mTNFR. Furthermore, the ELISA assay shows that the 3B11 potently inhibited mTNFα binding to mTNFR. This study may provide the basis for the TNFα blockers and diagnostics applications.

## 1. Introduction

Owing to high affinity and specificity, monoclonal antibodies have been of common use for several decades for a plethora of biotechnological and biomedical applications [1,2,3]. However, their drawbacks, such as their large size (150 kDa) or cost-ineffective production, limit their application in underdeveloped areas [4,5,6,7]. In 1995, a novel immunoglobulin isotype, namely, the immunoglobulin new antigen receptor (IgNAR), was found in cartilaginous fish [8]. Like the heavy-chain antibody (HcAb) found in Camelidae (camels, llamas, and their relatives) [9], IgNAR is also a homodimer of IgH chains that do not associate with IgL chains [8]. There are also many subtle varieties in IgNAR among different species of sharks. In nurse sharks, the variable domain of the new antigen receptor (VNAR) is joined to five constant (C1–C5) domains [10], while in whitespotted bamboo sharks, the C1 domain is spliced directly to C4 [11].

The VNAR domain, an Ig superfamily domain with four framework regions (FR1-4), has two β sheets associated together through two canonical cysteine residues in framework regions FR1 and FR3 [12,13]. In addition to framework regions, VNAR also contains two hypervariable regions (HVRs) and two complementarity-determining regions (CDRs), known as CDR1, hypervariable loop 2 (HV2), hypervariable loop 4 (HV4) and CDR3 [14,15], respectively. Besides these canonical cysteines, CDR3 can have one or two additional cysteines forming extra disulfide bridges within the VNAR domain. According to the position and number of non-canonical cysteines in the VNAR domain, IgNARs are classified into four types. Type I VNAR, which has only been found in nurse sharks (*Ginglymostoma cirratum*), possesses two non-canonical cysteine residues in CDR3 that form two disulfide bridges with FR2 and FR4 [8,16,17,18]. Type II VNAR contains the protruding CDR3, produced by a disulfide bridge formed between cysteine residues in CDR1 and CDR3 and possesses special paratopes that bind to pockets and grooves epitope [19,20,21,22]. Type III VNAR is similar to Type II, but with one highly conserved tryptophan residue in CDR1 positioned close to the disulfide bridge [16]. Unlike Type I–III VNARs, there is no non-canonical disulfide bond in Type IV [21,23].

Compared with conventional IgG, shark VNAR domains have advantageous properties due to their peculiar structure. First, sharks may generate high-affinity VNARs compared to conservative mammalian protein targets because of the evolutionary distance between mammals and sharks on the phylogenetic tree [24,25]. Second, the long CDR3 in sharks can access the buried epitopes or enzyme functional sites [24,25,26,27]. Third, VNARs may have good tissue penetration ability due to their small size [7,21]. Fourth, the structure of VNAR affords remarkable refolding properties after heat shock [28,29,30,31]. This makes them preferable in diagnostic applications and transport where heating may temporarily occur.

As a critically important inflammatory marker and drug target in sera, TNFα can be significantly induced after infection or injury via the activation of the immune cells [32,33]. On the one hand, TNFα plays a vital role in resolving infection and tissue repair through the signal transduction pathway [34]. On the other hand, sometimes, it may trigger a severe cytokine release storm that results in sepsis and autoimmune diseases, such as rheumatoid arthritis (RA), ankylosing spondylitis, psoriatic arthritis, and inflammatory bowel diseases (IBD) [35,36]. Until now, anti-TNFα monoclonal antibodies, soluble TNFα receptors with IgG chimeric protein, and anti-TNFα Fab fragments, which were used to block the bioactivity of TNFα in the inflammatory response, had become highly effective and powerful TNFα blocking agents in disease therapy [37,38,39,40]. Granted, each TNFα blocking strategy varies among studies in terms of both usefulness and effectiveness. The different intensity and tissue penetration achieved by different types of TNFα blockades may contribute to the variety of results. Furthermore, the cost of these agents remains a big challenge and limits their application in low-income families, primarily due to their large size, low expression level, and high immunogenicity [41].

Previous studies have isolated anti-TNFα, neutralizing VNARs through immunization of sharks [42,43,44]. However, there remains a significant requirement for new anti-TNFα blockades that may avoid or at least limit the drawbacks above. Extending diagnostic options for the growing clinical need is also desirable.

In this study, the mTNF recombinant protein was expressed and the whitespotted bambooshark was immunized with it. Then, an anti-mTNFα VNAR phage library was established through RT-PCR using mRNA from the whitespotted bambooshark PBLs. After the in vitro panning of the library against mTNFα antigens, 15 colonies of VNAR were identified. Interestingly, a new type of VNAR that targets mTNF was found. The 3B11 was expressed in the BL21 strain and ELISA and BLI were used to measure the affinity of 3B11. Additionally, the ELISA assay was used to test the 3B11 inhibition of TNFα-TNFR interaction. To better understand the mode of action, the 3D structures of VNARs were predicted using AlphaFold2 [45] and their binding to mTNFα was modeled through protein–protein docking, combining HawkDock [46] and RosettaDock [47].

## 2. Results

### 2.1. Isolation and Characterization of mTNFα Specific VNAR from Whitespotted Bambooshark

**Figure 1:**
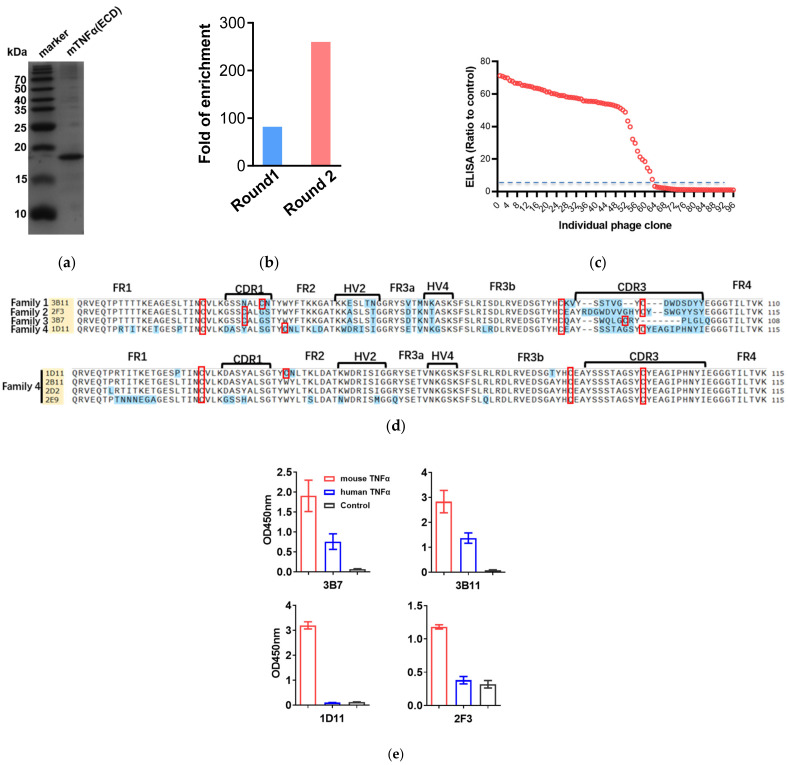
VNARs against mTNFα were selected by phage display panning. (**a**) SDS-PAGE analysis of mTNFα(ECD) purification by ProteinIso Ni-NTA resin. (**b**) The enrichment ratios of mTNFα-specific VNARs. (**c**) Independent clones (randomly picked from round 2) were tested for their ability to bind to mTNFα by phage display. 67% of the tested clones displayed a binding to mTNFα at least five times higher than their respective binding to non-fat milk. (**d**) Amino acid sequence alignment of anti-mTNFα VNARs. FR is framework region; CDR is complementarity-determining region; HV is hypervariable region. The Cys is indicated by a red box. (**e**) Cross-reactivity between four typical VNARs and hTNFα was detected by phage ELISA.

To obtain anti-mTNFα VNAR single domain antibodies, the extracellular domain of mTNFα (mTNFα ECD,77-235aa) was purified as an antigen with high purity through the *E. coli* expression system (Figure 1a). Total RNA was extracted from the PBLs after immunization of two whitespotted bamboosharks with the mTNFα(ECD) protein. The VNAR encoding gene fragments were then amplified by PCR with about 400 bp fragments purified with gel extraction, which were then inserted into pComb3XSS vectors and electroporated into TG1 cells. After obtaining the Anti-mTNFα VNAR phage-displayed library, the library capacity and the insert ratio were then determined. The correct insert ratio was about 100% through the colony PCR assay, and the library capacity reached approximately 1 × 10^9^ colony-forming units (CFU). Clone PCR analysis of 48 randomly chosen clones indicated that the percentage of the library insertion rate was 100% (Figure S1). Overall, these results indicate that an mTNFα-specific, phage-displayed VNAR library was successfully established.

Bio-panning was used to isolate mTNF-specific VNARs from a phage-displayed VNAR library. To evaluate the enrichment fold during phage display panning, the colony numbers among panning on mTNFα and non-fat milk as negative controls were compared. After two consecutive rounds of phage display bio-panning, the enrichment ratios of mTNFα-specific VNARs increased to about 260 times (Figure 1b). In addition, 63 positive clones with a binding ratio >5 were identified from a total of 96 randomly chosen clones by the phage-ELISA assay (Figure 1c). Then, the positive clones were sequenced, as shown in Figure 1d, and 15 anti-mTNFα VNARs with unique amino acid sequences were determined based on the sequencing analysis. Furthermore, these 15 anti-mTNFα VNARs were classified into 4 families based on the CDR3 amino acid sequences. Families 1–3 belong to type II VNAR. Interestingly, family 4 was a new type of VNAR that does not fit in any of the four known types (types I–IV). All 4 VNARs in family 4 possess one additional cysteine in the CDR3 domain but no cysteine in CDR1. In 1D11 VNAR, there is another cysteine in FR2. To further evaluate the species specificity of the VNARs, the human TNFα was expressed, and the phage ELISA was performed. As shown in Figure 1e, 3B7 and 3B11 could cross-react with human TNFα with weaker ELISA signals than mouse TNFα. However, 1D11 and 2F3 were specific for mouse TNFα, with no cross-reactivity toward human TNFα.

### 2.2. Expression of VNAR Single Domain Antibodies and 3B11 Antagonize the TNFR-TNFα Interaction

**Figure 2:**
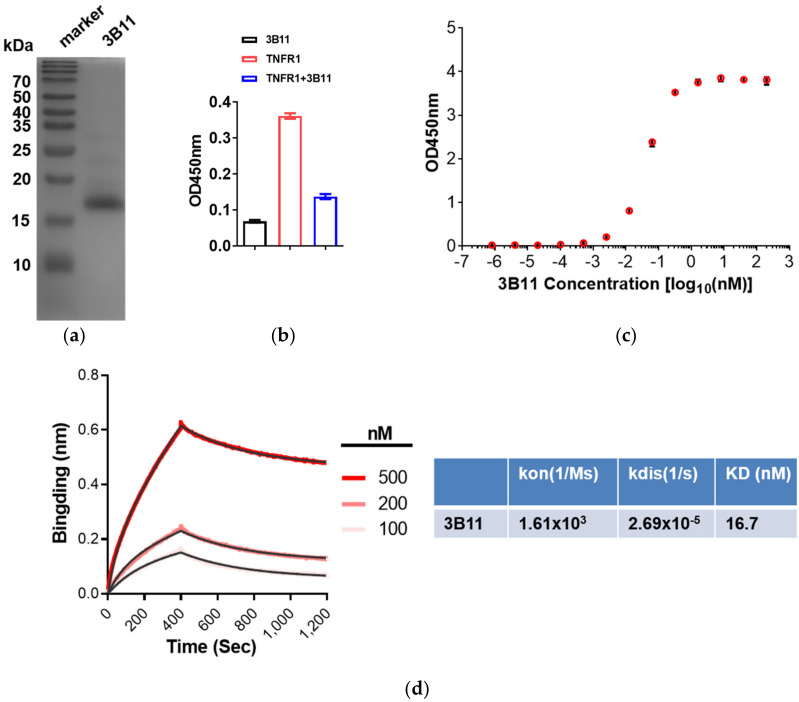
The binding affinity test of 3B11 VNAR and mTNFα. (**a**) SDS-PAGE and Coomassie blue staining of anti-TNFα 3B11 VNAR purification. (**b**) 3B11 VNAR potently blocked TNFR binding to TNFα by ELISA. (**c**) Binding affinities of anti-mTNFα 3B11 VNAR toward the mTNFα, as determined by ELISA. (**d**) Kinetics of the mTNFα -VNAR interaction determined by an Octet K2 BLI Analysis System.

To determine the VNAR single-domain antibodies binding kinetics, the four typical anti-mTNFα VNARs, 1D11, 2F3, 3B7 and 3B11, were produced in *E. coli* BL21(DE3) strain. The expression yield of 3B11 is about 2.16 mg from 200 mL *E*. *coli* culture, whereas the other three VNARs are expressed at extremely low levels. Nickel-charged HisTrap columns were used to purify 3B11 VNAR proteins from the *E. coli* supernatants. Sodium dodecyl sulfate polyacrylamide gel electrophoresis (SDS-PAGE) analysis showed good quality with more than 90% purity after one-step purification (Figure 2a). To test whether 3B11 inhibits the TNFα–TNFR interaction, the ELISA assay was performed and the result shows that 3B11 can potently block mTNFR binding to mTNFα (Figure 2b).

### 2.3. Affinity Assay of Anti-mTNFα 3B11 VNAR

To measure the affinity of the 3B11 VNAR with mTNFα, the ELISA and BLI experiments were performed. The ELISA EC_50_ value of 3B11 VNAR was 0.36 nM (Figure 2c). However, through the BLI assay, 3B11 had 16.7 nM KD binding affinity for mTNFα, which was much lower than the affinity tested by the ELISA result (Figure 2c,d). During the BLI assay, the SA biosensors were used to capture the biotin-labeled mTNFα through the biotin-NHS-biotinylation labeling reaction, which may cause the instability of the protein or the steric effect between mTNFα and VNAR.

### 2.4. Models of VNARs-mTNFα Complexes

**Figure 3:**
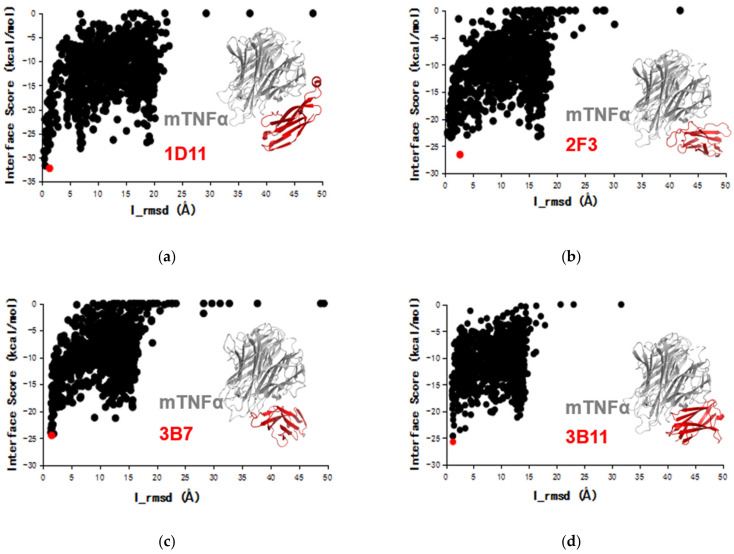
The Rosetta docking funnels of four VNARs 1D11, 2F3, 3B7 and 3B11 to mTNFα (**a**–**d**). The red plots have the lowest docking interface score (I-sc) with the interface root-mean-square deviation (I_rmsd) ≤ 4 Å. Inset: TNF trimer (gray) and VNARs (red).

**Figure 4:**
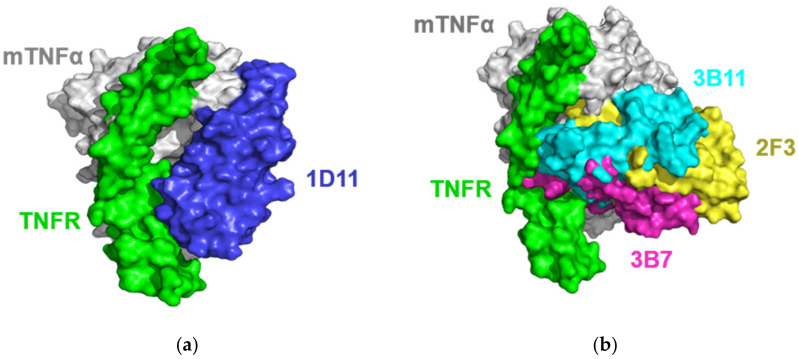
Surface representation of the four VNARs and the mTNFR (PDB ID: 6MKZ) on mTNFα. (**a**) 1D11 (blue) and mTNFR (green) on mTNFα (gray). (**b**) 2F3 (yellow), 3B7 (magentas), 3B11 (cyan) and mTNFR (green) mTNFα (gray).

In this work, the ideal docking funnels of the four VNARs binding to mTNFα were obtained (Figure 3). For each complex, the structure with the lowest I-sc and I-rmsd ≤ 4 Å from the docking trajectory was selected for further analysis. As shown in Figure 3, 1D11, 2F3, 3B7 and 3B11 attach to the concave surface of mTNFα. To further understand the mechanism of action, the structure of mTNFα in complex with mTNFR was constructed using the hTNFα-hTNFR complex structure [48] as a reference. As a result, Figure 4 shows that all four VNARs’ epitopes overlapped in part with that of mTNFR. In detail, 1D11 occupies the bottom of the receptor’s binding area on mTNFα (Figure 4a), and 2F3, 3B7 and 3B11 occupy the central area of the receptor’s binding area on mTNFα (Figure 4b). However, 3B7 has the largest overlapped area with the receptor compared to other VNARs.

## 3. Discussion

The whitespotted bamboo shark is a member of the hemiscyllidae family of sharks, which is commonly found in the coral reefs of the Pacific Ocean. They are widely distributed in the coastal areas of southeastern China and surrounding waters and are also used for human consumption. Previously, the nurse shark was the most commonly used shark species for immunization and VNAR preparation. Comparably, the whitespotted bambooshark, which is relatively small and easy to keep, is an attractive alternative animal model to study and obtain specific antigen VNARs through immunization. In this study, the mouse TNFα protein was used to immunize whitespotted bamboosharks. Previously, many studies combined subcutaneous and intravenous administration for shark immunization in different sharks [49,50,51,52]. In this work, whitespotted bamboosharks were immunized following the subcutaneous and intravenous administration immunization protocol. TNFα-specific VNARs were obtained through phage display panning, which indicated that the immunization protocol is effective for high-affinity VNAR isolation in whitespotted bamboosharks. 

However, after immunization, there was no secondary antibody available for the IgNAR from whitespotted bamboosharks. The commercial horn shark IgNAR antibody from GeneTex (GTX128445) was tested through Western blot and ELISA, but this IgNAR antibody does not work for the IgNAR from whitespotted bamboosharks. In the future, the secondary antibodies/nanobodies targeting IgNAR from whitespotted bamboosharks, which make it feasible to test the IgNAR titer in whitespotted bamboosharks, will be required.

Single-domain antibodies are becoming a promising tool both in diagnostics and therapeutic applications. In this study, the mTNFα VNARs were isolated through immunization of whitespotted bamboosharks and phage display panning. Among the VNAR sequences, a very special VNAR type was identified. In this new kind of VNAR, 1D11 has two non-canonical cysteine residues in FR2 and CDR3, but through the AlphaFold2 prediction, the two cysteines do not form a disulfide bridge. There is only one non-canonical cysteine in the other 3 members of this type of VNAR. To the best of our knowledge, this is the first report on this kind of VNAR that targets specific antigen, and more studies are needed to clarify the characteristics of this new kind of VNAR—for example, the epitope, thermostability, affinity and so on.

In this research, the affinity of the VNAR 3B11 was measured through ELISA and BLI, both of which show that 3B11 has a high affinity to mTNFα. Moreover, the molecular mimicry of anti-mTNFα VNARs indicate that all the four typical VNARs bind to the mTNFα epitopes that partially overlapped with TNFR, which means that all the VNARs may be potential mTNFα blocking agents. So, these mTNFα potential binders may be very good tools for the research of TNFα in the mouse model.

## 4. Materials and Methods

### 4.1. Ethics Statement

All procedures were approved by the Animal Care and Use Committee of the Third Institute of Oceanography, Ministry of Natural Resources, and conformed to the guidelines of the Fujian Provincial Department of Science and Technology for the Administration of Affairs Concerning Experimental Animals. 

### 4.2. mTNFα Protein Expressing

The mTNFα (77-235 aa) DNA sequence was cloned into a pET-28a (+) vector using *BamH*I and *Xho*I restriction enzymes (Takara, Beijing, China). The plasmid was transformed into *Escherichia coli* BL21 (DE3) cells and plated on LB-Agar (1% tryptone, 0.5% yeast extract, 1% NaCl, 1.5% agar) with 30 µg/mL Kanamycin sulfate at 37 °C overnight. Single colonies were selected and cultured in LB media. When OD600 reached 0.4–0.6, the culture was induced with 1 mM IPTG and incubated at 30 °C for 16 h. Cells were harvested and washed twice with a PBS buffer (Sangon, Biotech, Shanghai, China). Then, the cells were lysed by Ultrasonic machining in a lysis buffer (25 mM Tris, 150 mM NaCl, 1 mM PMSF, 1 mg/mL lysozyme, 10% glycerol, pH 7.5). Protein extracts were collected by centrifugation at 12,000 rpm for 30 min. The mTNFα containing His-tag was purified by ProteinIso Ni-NTA resin (TransGen Biotech, Beijing, China) and natively eluted with a different imidazole buffer (25 mM Tris, 150 mM NaCl, 25–500 mM imidazole, 10% glycerol, pH 7.5). The eluted mTNFα protein was subsequently dialyzed with a dialysis buffer (1 × PBS, pH 7.5), and a portion of the mTNFα protein was treated by thrombin protease overnight to remove His-tag. 

### 4.3. Immunization of Whitespotted Bamboo Sharks

Briefly, as shown in Table 1, two whitespotted bamboo sharks weighing about 0.5 kg were immunized, according to the protocol modified from previous studies. The mTNFα protein was emulsified in equal amounts of adjuvant (CFA (complete Freund’s adjuvant) or IFA (incomplete Freund’s adjuvant)) and subcutaneously injected in the pectoral fin as a mixed antigen cocktail. Subsequent boosts were administered intravenously in the caudal vein. Two weeks after the fourth immunization, the sharks’ PBLs were isolated to construct an immune VNAR library. 

The whitespotted bamboo sharks were comfortably housed at the Third Institute of Oceanography, Ministry of Natural Resources, and were anesthetized with MS-222 (Sigma, St. Louis, MO, USA) at approximately 0.1% (*w*/*v*) in artificial seawater before any procedure.

### 4.4. VNAR Library Construction

By the TRIZOL method, total mRNA was extracted from the purified PBLs and the concentration was measured by optical density at 260 nm. Then, cDNA was synthesized using PrimeScript™ II 1st Strand cDNA Synthesis Kit (Takara, Beijing, China). The VNAR fragments were obtained after PCR amplification of the cDNA by PCR using specific primers (Table 2). The PCR products corresponding to VNAR genes were analyzed by agarose gel electrophoresis. At the same time, the PCR products were digested with the SfiI restriction enzyme (NEB, Ipswich, New England), then inserted into the phagemid pComb3XSS with T4 ligase (Thermo Scientific, Waltham, MA, USA). By electroporation, recombinant plasmids were transformed into *E. coli* TG1 cells and plated onto 2 × YT-Agar (1.6% tryptone, 1% yeast extract, 0.5% NaCl, 1.5% agar, 1% glucose) containing 100 μg/mL ampicillin and cultured at 37 °C overnight. 

### 4.5. Selection of TNFα-Specific VNAR by Phage ELISA

The Immuno-tubes (Thermo Scientific, Waltham, MA, USA) coated with 5 mL of mTNFα with respective concentrations of 100 µg/mL (Round 1) and 50 µg/mL (Round 2) were incubated overnight at 4 °C. After washing and blocking by 5% Not-fat Powdered Milk (Sangon Biotech, Shanghai, China), 100 μL of the amplified phage display library (3.54 × 10^13^ pfu/mL) was added to each immune-tube and incubated for 1 h at room temperature in a rotator. The unbound phage was removed by washing 3 times with PBST (PBS, 0.1% Tween-20) in Round 1. The number of washes was increased to 6 times for subsequent rounds of panning. Two rounds of panning were performed. Phagemid particles were eluted using 0.1 M HCl, which was neutralized by adding 1 M Tris-base, and immediately reinfected with *E. coli* TG1. After 1 h of incubation on a shaker, the cell was plated onto a 2 × YT containing 1% glucose and 100 μg/mL ampicillin and cultured at 37 °C overnight. 

A total of 96 *E. coli* clones were randomly selected, and the phage supernatant containing the VNAR fragments were obtained by the phage display technique. The mTNFα was dissolved in a PBS buffer at 10 μg/mL to coat 96-well plates, 100 μL/well, at 4 °C overnight. The irrelevant antigen used was 5 μg/mL BSA in PBS. After the plate was blocked with 5% Non-fat Powdered Milk in the PBS buffer, 100 μL phage supernatant was added to the plate. Binding was detected by an HRP conjugated mouse anti-M13 antibody (Sino Biological, Beijing, China). The cut-off value for the positive binder was set as a 5× higher signal compared to the control. 

### 4.6. Soluble VNAR Production and Purification

According to the DNA sequencing results, VNAR binder sequences were cloned into a pET-28a (+) and were transformed into *E. coli* BL21 (DE3) cells. The form colonies were pooled in 500 mL LB media containing 30 μg/mL Kanamycin at 37 °C until the OD600~0.4–0.6. The culture was induced with 0.1 mM IPTG and incubated at 16 °C overnight for soluble protein production. Bacteria pellets were spun down and resuspended in 30 mL of ice-cold lysis buffer. The cell suspension was incubated on ice for 30 min. Cells were ultrasound broken on ice. The slurry was then centrifuged at 12,000 rpm for 30 min at 4 °C. Soluble VNAR containing His-tags was purified from the cell lysate by Ni-NTA resin and finally used different density imidazole buffers to elute VNAR protein.

### 4.7. ELISA for VNAR Affinity Detection

Antigens were coated onto a 96-well ELISA plate (NEST, Wuxi, China) at an amount of approximately 100 ng per well in the PBS buffer overnight at 4 °C. The well surface was then blocked with a blocking buffer (PBS, 5% Not-fat Powdered Milk) at 37 °C for 2 h. Antigens coated reference 4.7. For the VNAR affinity test, the scramble VNAR that does not bind the mTNFα was used for negative controls. The VNAR was serially 5-fold dilution from 1 μM to 0.8192 fM in the blocking buffer. After 2 h of incubation with VNAR at room temperature, His-tag mAb (Bioword, Nanjing, China) was diluted at 1:1000 and incubated for 2 h at room temperature again. Then, the goat anti-mouse lgG (Abclonal, Wuhan, China) was diluted at 1:5000 and incubated for 1 h at room temperature. Nine washes with PBST were carried out between each incubation to remove nonspecific absorbances. After the final wash, the samples were further incubated in dark with a freshly prepared TMB Single-Component Substrate solution (Solarbio, Beijing, China) for 10 min at room temperature to develop the signals. After the stop solution (92 mM H_2_SO_4_), the plates were read at 450 nm on a plate reader. The raw data were processed by Prism 7I (GraphPad, San Diego, CA, USA) to calculate EC50. For 3B11 blocking TNFα-TNFR interaction assay, mTNFα were coated onto a 96-well ELISA plate, then 50 µL TNFRSF1AhFc (20 ng/mL, Sino Biological, Beijing, China, Cat:50496-M02H) and 50 µL 3B11 (3 μM) was successively added to the wells. Control wells were added with 100 μL using 5% milk in PBST. After a 2 h incubation, the Goat Anti-Human IgG Fc (HRP) (Abcam, Cambridge, UK) was diluted at 1:5000 and incubated for 1 h at room temperature. The following procedure is the same as above.

### 4.8. BLI-Based Affinity Assay

BLI-based mTNFα binding inhibition experiments were carried out by BLI using an Octet K2 Protein Analysis System. The measurements were performed using Streptavidin (SA) biosensors. In brief, the mTNFα proteins were immobilized onto the SA biosensor surface via biotin (biotin-NHS)-biotinylation (MCE, Shanghai, China) labeling reaction, following the manufacturer’s directions. The mTNFα proteins were labeled with biotin at a concentration of 1 μM for half an hour and dialyzed with PBS at 4 °C overnight. All steps were performed at room temperature, with a working volume of 200 µL in each well. The mTNFα antigen–antibody basic kinetic experiments were made up of baseline (PBST, 1 × PBS + 0.02% tween-20, 60 s); mTNFα protein loading (2 µg/mL mTNFα, PBST, pH7.4, 300 s); baseline2 (PBST, 120 s); 500 nM, 250 nM, 100 nM, 50 nM anti-mTNFα VNAR association (anti-mTNFα VNAR, PBST, 400 s); and dissociation (PBST, 800 s). For the control sample, the same amount of PBST buffer was added to the mTNFα sample to remove the interference from the PBST buffer itself. The response data were normalized using Octet data analysis studio 12.2 (Sartorius, Goettingen, Germany). 

### 4.9. Computational Modeling

For each VNAR, the multiple sequence alignment file was first generated using MMseqs2 [53]. Then, the 3D structures of 3B7, 3B11, 1D11 and 2F3 were predicted by AlphaFold2 [45], and the top-ranked models with high pLDDT were selected as their 3D structures.

Based on the predicted 3D structures of VNARs and the crystal structure of mTNFα (PDB ID: 2TNF [54]), the binding modes between VNARs and mTNFα were obtained by using the protein–protein docking strategy combining HawkDock [46] and RosettaDock [47]. First, HawkDock [46] was performed to preliminarily explore the poses of each VNAR and TNFα, which generated the top 10 prediction complex models. Referring to the binding epitopes of Nanobodies on human TNFα [48], 5 reasonable models were then selected for RosettaDock using the same setup as recent studies [55,56]. In brief, the initial model was prepacked and used as a starting point for several rounds of local docking to generate 1000 decoys by running RosettaDock with the Monte Carlo (MC) refinement method [57]. Finally, the docking funnel of the trajectory describing the characteristics of the interface score (I-sc) of each decoy and the interface root-mean-square deviation (I_rmsd) was made to find the reasonable model of each complex [55,56,58].

### 4.10. Statical Analyses

For the biopanning and ELISA assays, data were analyzed using GraphPad Prism 7 (GraphPad, San Diego, CA, USA).

## 5. Conclusions

In this study, the ability to raise the specific IgNAR from whitespotted bamboosharks against mTNFα by immunization was demonstrated. After immunization, a phage display VNAR library was successfully constructed by PCR amplification. Selection from this phage display VNAR library resulted in 15 unique clones with specificity for mTNFα, suggesting that the enrichment of affinity VNARs against mTNFα proteins has been successfully achieved through iterative biopanning. Interestingly, one new type of VNAR targeting mTNFα that does not belong to any known VNAR type was identified. Additionally, 3B11 VNAR was expressed in *E. coli* and the binding affinity of 3B11 VNAR reached 16.7 nM through the BLI assay. The models of VNARs–mTNFα complexes were predicted by computational modeling combining HawkDock and RosettaDock. The results indicated that the four VNARs’ epitopes overlapped in part with that of mTNFR.

## Figures and Tables

**Table 1:** Schedule for immunization of whitespotted bamboo sharks with mTNFα.

Week Number	Procedure	Details	Immunization Route
0	Immunization 1	200 μg mTNFα in CFA	Subcutaneous
4	Immunization 2	200 μg mTNFα in IFA	Subcutaneous
8	Immunization 3	100 μg mTNFα soluble	Intravenous
12	Immunization 4	100 μg mTNFα soluble	Intravenous

**Table 2:** Details of primers used in the PCR experiment. SfiI restriction enzyme sites are underlined.

Primer	Sequence
VNAR-1- Forward	TCGCTACCGT ggcccaggcggcc CAACGGGTTGAACAAACACC
VNAR-2- Forward	TCGCTACCGT ggcccaggcggcc GCATGGGTTGAGCAAACACCG
VNAR-1- Reverse	TGATGGTGCT ggccggcctggcc TTTCACAGTCAGAATGGTGC
VNAR-2- Reverse	TGATGGTGCT ggccggcctggcc TTTCACTGTTAGAAAAGTGCC

## Supplementary Materials

The following supporting information can be downloaded at: https://www.mdpi.com/article/10.3390/md20050307/s1. Figure S1: The PCR verified the library quality.
