# Supplementary figures and images for: Identification of Anti-TNFα VNAR Single Domain Antibodies from Whitespotted Bambooshark (Chiloscyllium plagiosum)
Source: Mar Drugs. 2022 Apr 29;20(5):307. doi: 10.3390/md20050307 (PMC9146136; doi:10.3390/md20050307)

**Supplementary Materials:**

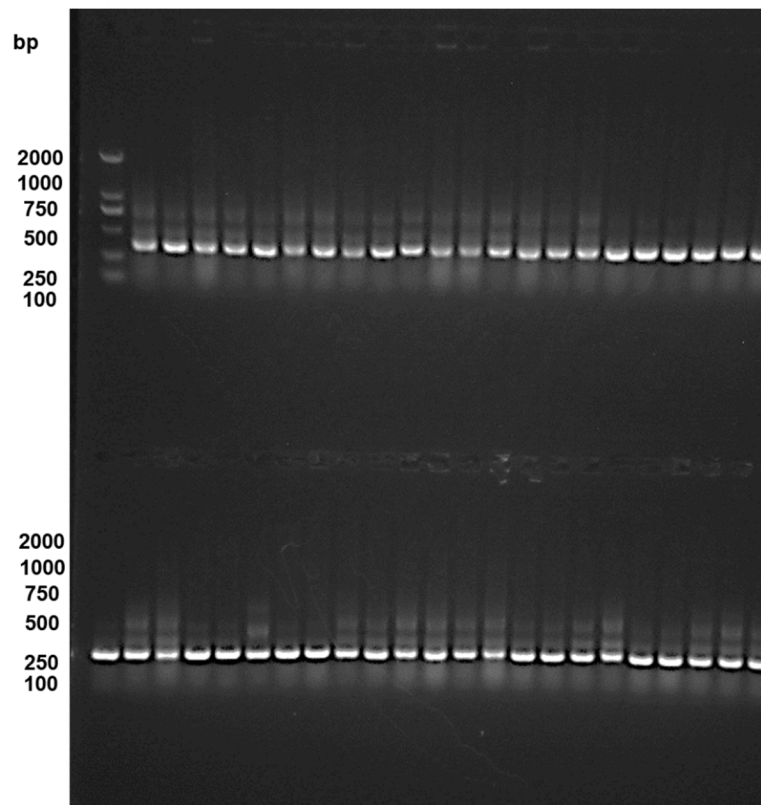

Figure S1: The PCR verified the library quality.

Supplement: Supplementary file 1 [file marinedrugs-20-00307-s001.zip › marinedrugs-1681943-supplementary.pdf]
